# Point-of care lung ultrasound in the NICU: uses and limitations of a new tool

**DOI:** 10.1186/1824-7288-40-S2-A25

**Published:** 2014-10-09

**Authors:** Francesco Raimondi, Fiorella Migliaro, Angela Sodano, Claudio Veropalumbo, Angela Carla Borrelli, Silvia Lama, Gianfranco Vallone, Letizia Capasso

**Affiliations:** 1 Division of Neonatology, Section of Pediatrics, Department of Medical Translational Sciences, Università “Federico II” , Naples, Italy; 2 Section of Radiology, Department of Advanced Biomedical Science, Università “Federico II”, Naples, Italy

## 

Pulmonary imaging in the neonatal intensive care unit (NICU) relies traditionally on the conventional chest radiogram. Translating evidences from adult emergency medicine, pediatricians and neonatologists have recently started to apply lung ultrasonography to the critical infant and child with respiratory problems [1].

**Figure 1:**
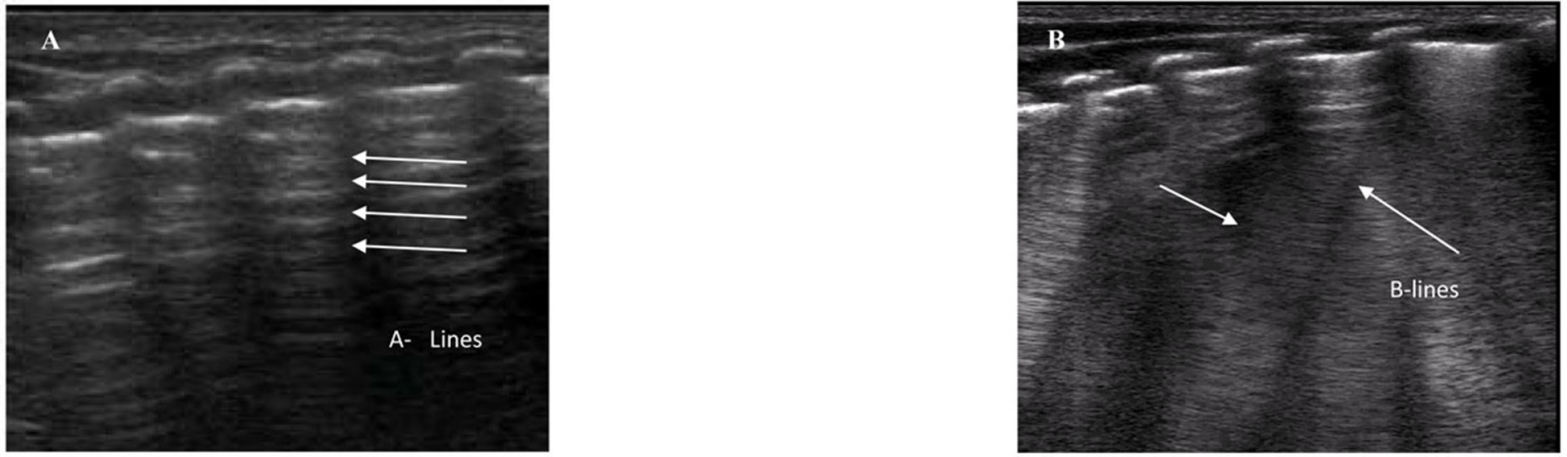
1A: reverberations of the pleural image (aka A-lines) in the normally aerated lung. 1B: the prevalence of vertical B-lines (in between arrows) has been linked to the interstitial syndrome in the adult and to a progressive aeration of the neonatal lung after birth.

Because of the high impedance of a normally aerated lung, an ultrasound scan does not render an anatomical image of the organ. However, ultrasounds clearly define the pleural surface with the normal sliding movement. Pleural effusions and lung consolidations can also be reliably diagnosed with ultrasonography. However, ultrasounds penetrating the lung will also generate artifacts (i.e. structures not naturally present in the living that appear as authentic images). These imagery anomalies come from the machine acquisition of the ultrasound beam path through means with markedly different acoustic impedance in close proximity. The horizontal reverberations of the pleural line (aka the A lines - see Figure 1A) and the vertical hyperecoic image departing from the pleura (aka the B lines- see Figure 1B) are commonly seen artifacts.

Real and artefactual images have been combined in disease specific ultrasound profiles. Using these profiles, adult emergency physicians have shown that lung ultrasound outperforms conventional radiology in relevant diagnoses such as pleural effusion, pneumonia or pneumothorax.

Pediatricians have started to use lung ultrasound with success to their patients affected by pneumonia but also by bronchiolitis [2]. In the NICU, lung ultrasound has found its specific applications, not without controversies [3]. Transient Tachypnea of the Newborn and Respiratory Distress Syndrome have been described with ultrasound profiles that are both highly sensitive and specific [4]. A relevant limitation of chest ultrasound is that surfactant administration gives a persistent white lung image rendering any follow-up essentially unfeasible. Ultrasounds can, however, accurately describe the fluid to air transition after birth and identify those neonates who will fail to adapt to extrauterine life needing respiratory support [5]. In a series of preterm neonates with moderate respiratory distress, recent work by our group shows that chest ultrasound is significantly more accurate than conventional radiograph in predicting the failure of non invasive ventilation [6].

Lung ultrasound is a very promising clinical tool in the NICU whose potential applications are well worth future multicenter trials.
